# Knee osteoarthritis in young growing rats is associated with widespread osteopenia and impaired bone mineralization

**DOI:** 10.1038/s41598-020-71941-8

**Published:** 2020-09-15

**Authors:** Supitra Namhong, Kannikar Wongdee, Panan Suntornsaratoon, Jarinthorn Teerapornpuntakit, Ruedee Hemstapat, Narattaphol Charoenphandhu

**Affiliations:** 1 Department of Physiology, Faculty of Science, Mahidol University, Bangkok, Thailand; 2 Center of Calcium and Bone Research (COCAB), Faculty of Science, Mahidol University, Rama VI Road, Bangkok, 10400 Thailand; 3 Faculty of Allied Health Sciences, Burapha University, Chonburi, Thailand; 4 Department of Physiology, Faculty of Medical Science, Naresuan University, Phitsanulok, Thailand; 5 Department of Pharmacology, Faculty of Science, Mahidol University, Bangkok, Thailand; 6 Institute of Molecular Biosciences, Mahidol University, Nakhon Pathom, Thailand; 7 The Academy of Science, The Royal Society of Thailand, Bangkok, Thailand

**Keywords:** Physiology, Bone

## Abstract

Osteoarthritis (OA) leads to joint pain from intraarticular inflammation with articular cartilage erosion, deterioration of joint function and abnormal subchondral bone structure. Besides aging, chronic repetitive joint injury is a common risk factor in young individuals. Nevertheless, whether OA is associated with bone loss at other skeletal sites is unclear. Since OA-associated proinflammatory cytokines—some of which are osteoclastogenic factors—are often detected in the circulation, we hypothesized that the injury-induced knee OA could result in widespread osteopenia at bone sites distant to the injured knee. Here we performed anterior cruciate ligament transection (ACLT) to induce knee OA in one limb of female Sprague–Dawley rats and determined bone changes post-OA induction by micro-computed tomography and computer-assisted bone histomorphometry. We found that although OA modestly altered bone density, histomorphometric analyses revealed increases in bone resorption and osteoid production with impaired mineralization. The bone formation rate was also reduced in OA rats. In conclusions, ACLT in young growing rats induced microstructural defects in the trabecular portion of weight-bearing (tibia) and non-weight-bearing bones (L5 vertebra), in part by enhancing bone resorption and suppressing bone formation. This finding supports the increasing concern regarding the repetitive sport-related ACL injuries and the consequent bone loss.

## Introduction

Osteoarthritis (OA)—a major cause of joint pain and disability worldwide—is characterized by the progressive erosion of articular cartilage, with concomitant structural and functional changes of tissues around the joints, including synovium, meniscus, periarticular ligaments and subchondral bone^1^. It often affects lower limb joints, e.g., knee, hip, foot, and ankle^1^. Local inflammation is an important feature of OA, as indicated by overproduction of proinflammatory cytokines such as interleukin (IL)-1β, IL-6, IL-8, and tumor necrosis factor (TNF)-α^2^. A spillover of these cytokines into the circulation can cause systemic inflammation that might lead to osteoclastogenesis and bone erosion^3^.

The prevalence of OA increasingly parallels with an increase in the number of people aged ≥ 60 years^1^. Several factors are associated with OA risk, e.g., age, gender (female-to-male ratio > 1), obesity, genetic, endocrine injury, and joint trauma/sport injuries^1,2^. Acute or repetitive injuries especially in the lower limb joints due to exercise or sports, e.g., football, basketball, and skiing, can cause OA^1,4^. Most of the injuries involve the anterior cruciate ligament (ACL), and such repetitive injuries can increase the risk for developing knee OA, especially in women who have a higher incidence of ACL injury and OA^5,6^. In addition to joint rupture, local bone loss has been reported in the bones around the injured knee as indicated by a decrease in bone mineral density (BMD) and impaired bone microstructure, e.g., decreased trabecular thickness with increased trabecular separation in femur and tibia^7,8^. However, an association between OA and systemic bone loss at the other sites, e.g., vertebral bone loss, has not been addressed. It has been documented in humans that knee OA was correlated with vertebral bone loss/osteoporosis, but this correlation has not yet been confirmed^9,10^.

Although bone change in aging-related OA is well-known, OA-induced trabecular bone change in tibia and vertebra are less documented, especially during the growing period. We hypothesized that early OA in young growing rats could weaken trabecular bone microstructures, which, in turn, compromise bone strength. Therefore, the present study aimed to investigate changes in the tibial and vertebral bone microstructure in rats with ACL injury-induced OA. The present OA model has been reportedly associated with significant elevation of local factors, such as prostaglandin E_2_ (PGE_2_), and systemic osteoclastogenic cytokines, such as IL-1β, IL-6, and TNF-α^11–13^.

## Materials and methods

### Experimental design

**Figure 1:**
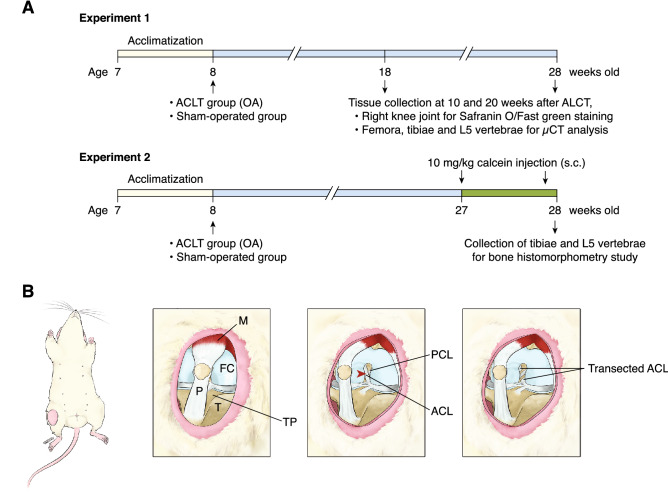
(**A**) Diagrams show experimental timelines of anterior cruciate ligament transection (ACLT)-induced knee osteoarthritis (OA) model (please see details in the methods). (**B**) An artwork demonstrates ACLT surgery performed on the right knee joint. Red arrow head indicates the point of incision. *ACL* anterior cruciate ligament, *FC* femoral condyle, *M* quadriceps femoris muscle, *P* patellar tendon, *PCL* posterior cruciate ligament, *T* Tibia, *TP* tibial plateau.

This study consisted of two experiments (Fig. 1A). In the first experiment, after 7-day acclimatization, rats were randomly divided into 2 groups, i.e., sham-operated and OA groups, and sub-divided into 10- and 20-week post-operation groups (n = 7 per group). In the OA group, rats were subjected to anterior cruciate ligament transection (ACLT) on their right knee joint, whereas in the sham-operated group, the rats were under the surgical methods similar to ACLT, except that ACLT transection was omitted. At 10 and 20 weeks after operation, right knee joints were collected for histopathological assessment of OA; however, some specimens were not in good orientation for OA grading (only 5 specimens/group were used for grading). Moreover, femora, tibiae and L5 vertebrae were then collected for volumetric BMD analysis by micro-computed tomography (μCT). In the second experiment, bone microstructural changes were studied in sham-operated and OA groups (n = 7 per group) only at 20 weeks post-operation. On day 6 and day 1 prior to euthanasia, rats were subcutaneously injected with 10 mg/kg body weight calcein (Sigma) to label bone. Bone microstructural changes in the tibiae and L5 vertebrae were analyzed by static and dynamic bone histomorphometry. The sample size was calculated as described by Malone et al.^14^.

### Anterior cruciate ligament transection (ACLT)-induced knee OA

The ACLT operation was performed on the right knee joint to induce mild knee OA (Fig. 1B), as previously described^15,16^. Briefly, rats were anesthetized by inhaling 5% isoflurane for induction (Attane, Piramal Critical Care, Bethleham, PA, USA) and anesthesia was maintained by 2% isoflurane. The rats received injection of 20 mg/kg bodyweight antibiotic cefazolin sodium (Cefaben; L.B.S. Labroratory, Bangkok, Thailand) and 2 mg/kg body weight tramadol hydrochloride (Tramadol-100, L.B.S. Laboratory). A 2-cm longitudinal incision was performed over the right distal patella, proximal to the tibial plateau. The joint capsule was incised with a sterile surgical blade, in which the patella was dislocated laterally to allow a greater access to the femorotibial joint. The fat pad over the intercondylar area was bluntly dissected to expose the ACL in the intercondylar region. Then, the ACL was completely transected with a micro-surgical blade. Successful transection of ACL was confirmed by the presence of anterior drawer sign. Finally, the joint capsule, subcutaneous layer and skin were sutured with vicryl 5-0 braided absorbable suture and silk 3-0 braided non-absorbable suture, respectively. For sham operation, the anesthesia and surgical procedure were similar except that the ACL was left intact. After surgery, the rats received daily injection of 20 mg/kg bodyweight antibiotic cefazolin sodium (Cefaben) and 2 mg/kg body weight tramadol hydrochloride (Tramadol-100) for 4 consecutive days to prevent post-operative infection and post-operative pain, respectively. They were daily monitored for 7 days for symptoms and signs of infection, e.g., alteration of consciousness, joint pain, immobility, bleeding, wound swelling or pus discharges, which were exclusion criteria of the present study.

### Histopathological assessment of OA

The right knee joints were collected and cleaned of adhering tissue, then fixed overnight in 0.1 M phosphate-buffered saline (PBS) containing 4% paraformaldehyde. The decalcification was performed by immersing the knee joints in 20% w/v ethylenediamine tetraacetic acid (EDTA; Loba Chemie Pvt. Ltd., Mumbi, India) at 4 °C for 6 weeks. Decalcifying solution was replaced every 3 days. Then, knee joints were dehydrated at room temperature through an ethanol series, cleared in xylene, embedded in paraffin, and cut longitudinally into 5-μm-thick sections. The paraffin sections were stained with Safranin O and Fast green for histopathological grading according to the modified method of the Osteoarthritis Research Society International (OARSI) osteoarthritis cartilage histopathology assessment system^17^. OA grade as defined by OA depth progression into the cartilage indicates severity of the osteoarthritic process. OA grades are divided into 7 levels from 0 to 6, the higher number indicates greater depth progression into the cartilage. Based on the osteoarthritis cartilage histopathology assessment system of the OARSI, Grade 0 indicates intact surface cartilage and articular morphology, whereas Grade 1 is the threshold for OA^17^. All paraffin sections were assessed at × 40 magnification using a light microscope (Olympus BX51, Tokyo, Japan).

### Computer-assisted bone histomorphometry

Trabecula-rich bones, i.e., proximal tibiae and L5 vertebrae, were cleaned of adhering tissues, and dehydrated in 70%, 95% and 100% v/v ethanol for 3, 3 and 2 days, respectively. Dehydrated tibial and L5 vertebral specimens were embedded in methyl methacrylate resin and polymerized resin at 42 °C for 48 h. Resin-embedded bone specimens were cut longitudinally by a microtome equipped with a tungsten carbide blade (model RM2265; Leica, Nussloch, Germany) to obtain 7-µm- and 12-µm-thick sections for the staining and unstaining histomorphometric techniques, respectively. For the staining technique, bone sections were stained with Goldner’s trichrome. The unstained bone sections were examined for the double lines of calcein labeling. Image analysis was performed under a fluorescent/light microscope (model eclipse Ni-U; Nikon) with the computer-assisted OsteoMeasure system version 4.10 (OsteoMetrics Inc., Atlanta, GA, USA). The region of interest covered trabecular microstructure at secondary spongiosa, which was analyzed to obtain static parameters [i.e., trabecular bone volume normalized by tissue volume (BV/TV; %), osteoid volume normalized by tissue volume (OV/TV; %), osteoid thickness (O.Th; µm), trabecular number (Tb.N; mm^–1^), trabecular thickness (Tb.Th; µm), osteoblast surface (Ob.S/BS; %), osteoclast surface (Oc.S/BS; %), and active erosion surface (ES/BS; %)] and dynamic parameters [i.e., double labeled surface (dL.S/BS; %), mineralizing surface (MS/BS; %), mineral apposition rate (MAR; µm/day), and bone formation rate (BFR/BS; µm^3^/µm^2^/day)] from stained and unstained sections, respectively.

### Micro-computed tomography (µCT)

For the ex vivo scanning to obtain vBMD (g/cm^3^), femora, tibiae and L5 vertebrae were wrapped with saline-moist gauze and scanned at 65 kV, current of 615 µA (Skyscan 1178 high-speed in vivo/ex vivo µCT; Bruker MicroCT, Kontich, Belgium). For femur and tibia, the region of interest (ROI), i.e., trabecular region, was 1.360–5.610 mm distal to the growth plate. The trabecular region of L5 vertebrae was analyzed at 1.275–4.675 mm distal to the growth plate. Rotation angle was 0.54° at each step and voxel size was 85 µm^3^ isotropically. The three-dimensional (3D) images were reconstructed by NRecon software (Skyscan, version 1.6.4.8). Serial 8-bit images were analyzed by CTAn software (version 1.14.4).

### Statistical analysis

Results are expressed as means ± SE. Unless otherwise specify, comparisons between two sets of data with normal distribution were performed by unpaired Student’s *t*-test. OARSI scores were tested by nonparametric Mann–Whitney test. The level of significance for all statistical tests was *P* < 0.05. Data were analyzed by GraphPad Prism 8 (GraphPad Software Inc., San Diego, CA, USA).

### Animals

Seven-week-old female Sprague–Dawley rats were obtained from the National Laboratory Animal Centre of Thailand, Mahidol University. The animals were housed in polystyrene cages (2 animals per cage), with ambient temperature of at 20–25 °C and 50–60% relative humidity. The animals were fed standard laboratory chow (CP Co., Ltd., Thailand) and reverse osmosis water ad libitum. The experimental protocol has been approved by the Institutional Animal Care and Use Committee (IACUC) of the Faculty of Science, Mahidol University. All experiments were performed in accordance with relevant regulations and the ARRIVE (Animal Research: Reporting of In Vivo Experiments) guideline.

## Results

**Figure 2:**
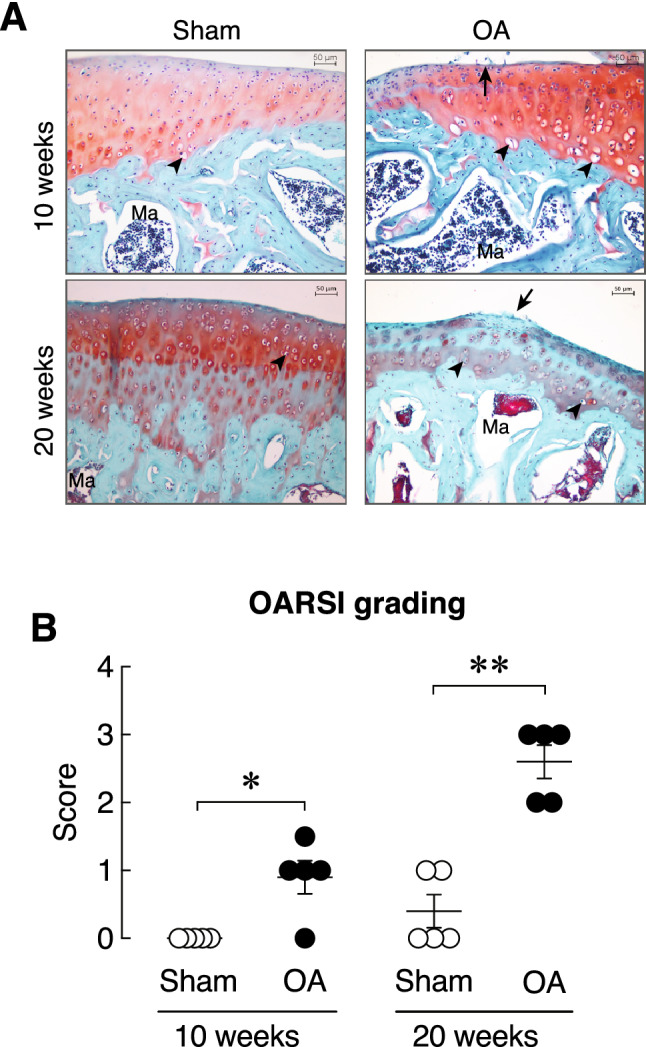
(**A**) Representative photomicrographs of right knee joint obtained from sham and OA rats at 10 and 20 weeks after OA induction stained with Safranin O/Fast green staining. Red color indicates articular cartilage. Green color indicates subchondral bone. Arrow indicates mild abrasion on the articular cartilage surface of tibial plateau. Arrow head indicates articular chondrocytes. Ma, marrow. Scale bars are 50 µm. (**B**) OARSI average grading score in the right knee joint obtained from sham and OA rats at 10 and 20 weeks after OA induction (*n* = 5 per group). *P < 0.05, **P < 0.01 vs. corresponding Sham group.

At week 10 after OA induction, the sham-operated knee joint exhibited intact smooth articular cartilage, i.e., smooth articular surface and appropriate chondrocyte orientation (Fig. 2A). On the other hand, OA joint showed mild abrasion on the surface of articular cartilage. Chondrocytes in the superficial layer were slightly distorted and some hypertrophic chondrocytes were observed. In Fig. 2B, the OA joint had a significantly higher OARSI grade of superficial articular cartilage layer than sham-operated joint (*P* < 0.01). At week 20 after OA induction, the sham-operated joint showed intact articular cartilage surface, whereas the superficial layer of the OA joint exhibited slight fibrillation with disoriented chondrocytes, some of which were hypertrophic. A significant loss of Safranin O-stained articular cartilage region was also observed in the OA joint. The 20-week OA joint had significantly higher OARSI grade than sham-operated knee joint. Thus, the damage of articular cartilages was aggravated during OA progression as expected, and ACLT in the present experiment effectively induced knee OA in rats at both week 10 and 20 post-OA induction.

**Figure 3:**
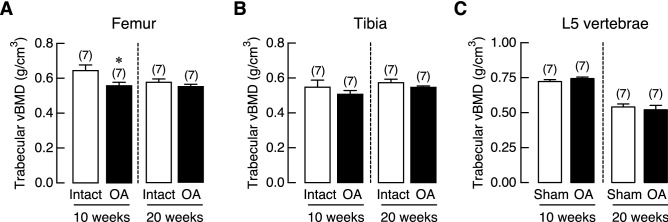
Trabecular volumetric bone mineral density (vBMD) of (**A**) femur, (**B**) tibia, and (**C**) L5 vertebrae in osteoarthritis (OA)-induced rats at 10 and 20 weeks after OA induction. Numbers in parentheses indicate numbers of animals. *P < 0.05 vs. corresponding OA intact leg.

**Figure 4:**
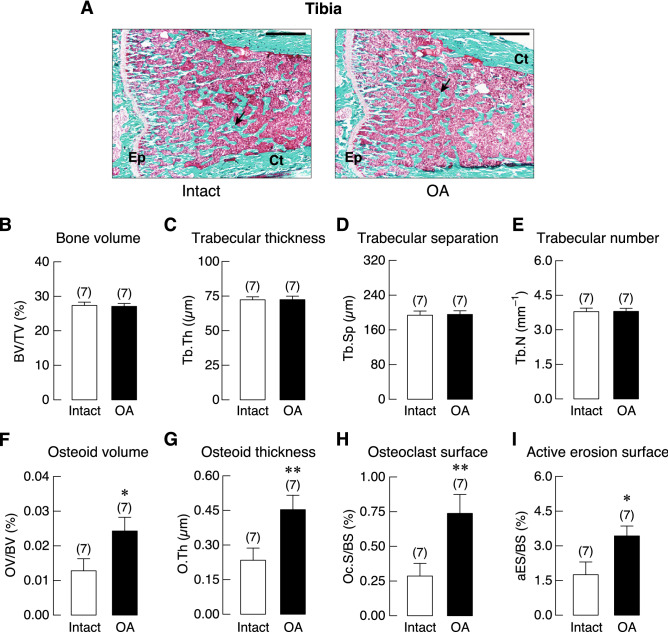
(**A**) Representative photomicrographs of Goldner’s trichrome stained tibial growth plate obtained from 20-week OA rat and its intact tibiae. Epiphyseal plate (Ep), and cortical shell (Ct) were identified. Mineralized trabeculae (*arrows*) and marrow cells were stained green and red, respectively. Bars, 1 mm. Microstructural analysis of tibial metaphysis in OA rats and its intact leg at 20 weeks after OA induction as determined by bone histomorphometry. (**B**) trabecular bone volume normalized by tissue volume (BV/TV), (**C**) trabecular thickness (Tb.Th), (**D**) trabecular separation (Tb.Sp), (**E**) trabecular number (Tb.N), (**F**) osteoid volume (OV) normalized by bone volume (BV), (**G**) osteoid thickness (O.Th), (**H**) osteoclast surface (Oc.S) normalized by bone surface (BS), and (**I**) active erosion surface (aES) normalized by BS. Numbers of animals in each group are shown in parentheses. *P < 0.05 and **P < 0.01 vs. intact leg.

**Figure 5:**
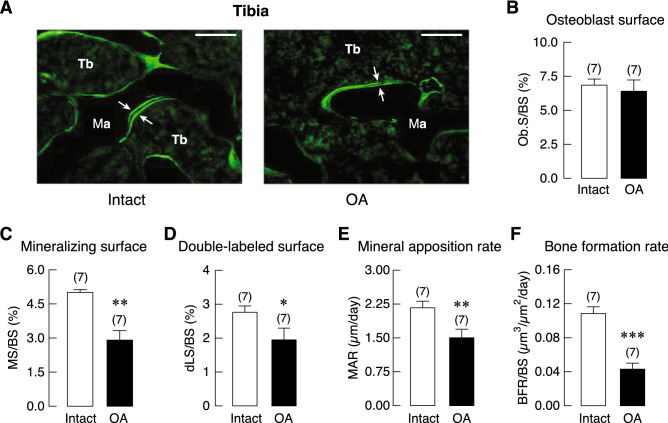
(**A**) Representative fluorescent photomicrographs of calcein labeling in the tibial metaphyseal trabeculae (Tb) in OA rat and its intact leg. Calcein as a calcium-chelating agent was injected into all rats at 6 and 1 days prior to tissue collection. Therefore, calcein signals on a bone-forming area appeared as double fluorescent lines (*arrows*), and the space between the two lines was the newly mineralized bone during the 5-day period. Ma designates marrow cavity. Bars, 100 µm. Bone formation-related parameters of tibial metaphysis in OA rats and its intact leg at 20 weeks after OA induction as determined by bone histomorphometry. (**B**) Osteoblast surface (Ob.S) normalized by bone surface (BS), (**C**) mineralizing surface (MS) normalized by BS, (**D**) double-labeled surface (dLS) normalized by BS, (**E**) mineral apposition rate (MAR), (**F**) bone formation rate (BFR) normalized by BS. Numbers of animals in each group are shown in parentheses. *P < 0.05, **P < 0.01 and ***P < 0.001 vs. intact leg.

Trabecular vBMD was assessed in femur and tibia of ACLT-operated knee joint (OA) and its contralateral side (Intact), as well as L5 vertebrae of sham-operated (Sham) and OA group. We found that OA had a tendency to decrease trabecular bone density in the femur rather than tibia. As shown in Fig. 3, a significant reduction of vBMD was observed only in the femur at 10 weeks after OA induction. Histopathological examination of the Goldner’s trichrome-stained tibial samples revealed that the trabecular bone in the subchondral area of OA group was lost. The trabecular plate became thinner with expansion of marrow space as depicted in Supplementary Fig. S1. Histomorphometric analyses of the tibiae revealed that 20-week OA induction did not affect bone volume (BV), trabecular number (Tb.N), trabecular thickness (Tb.Th), or trabecular separation (Tb.Sp). However, this induction period upregulated bone cell activities, as shown by the increases in osteoclast surface (Oc.S) and active erosion surface (aES) that reflected enhanced osteoclastic bone resorption, with increased osteoid thickness (O.Th) and osteoid volume (OV; Fig. 4). Moreover, osteoclast numbers (N.Oc) analyzed by using Goldner’s trichrome staining method in the tibiae of 20-week OA rats were greater than their intact tibiae (Supplementary Fig. S2). Osteoblast surface (Ob.S) did not change, while calcification process was distinctly impaired as indicated by reduction of mineralizing surface (MS), double-labeled surface (dLS), mineral apposition rate (MAR), and bone formation rate (BFR; Fig. 5).

**Figure 6:**
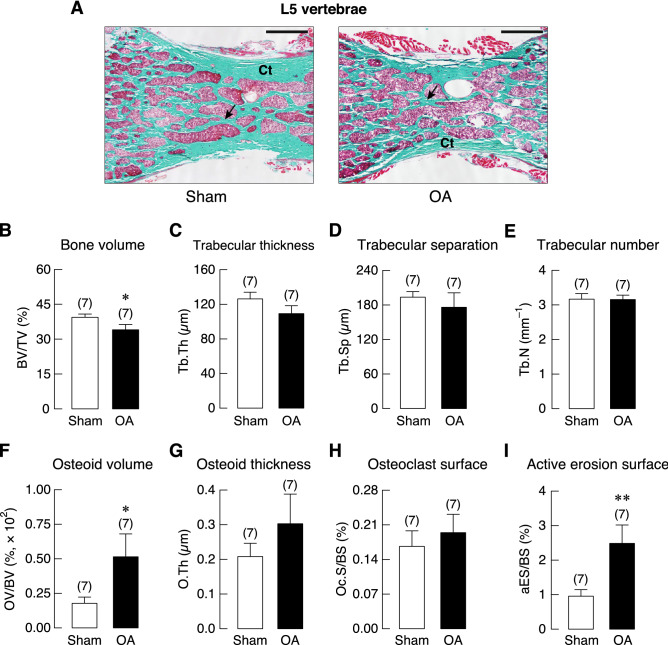
(**A**) Representative photomicrographs of Goldner’s trichrome stained L5 vertebrae obtained from 20-week OA and sham rats. Cortical shell (Ct) were identified. Mineralized trabeculae (*arrows*) and marrow cells were stained green and red, respectively. Bars, 1 mm. Microstructural analysis of L5 vertebrae in OA and sham rats at 20 weeks after OA induction as determined by bone histomorphometry. (**B**) trabecular bone volume (BV/TV), (**C**) trabecular thickness (Tb.Th), (**D**) trabecular separation (Tb.Sp), (**E**) trabecular number (Tb.N), (**F**) osteoid volume (OV) normalized by bone volume (BV), (**G**) osteoid thickness (O.Th), (**H**) osteoclast surface (Oc.S) normalized by bone surface (BS), and (**I**) active erosion surface (aES) normalized by BS. Numbers of animals in each group are shown in parentheses. *P < 0.05, **P < 0.01 vs. sham group.

**Figure 7:**
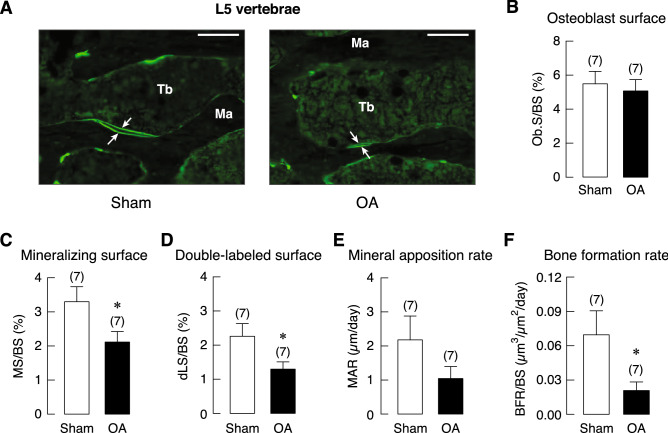
(**A**) Representative fluorescent photomicrographs of calcein labeling in the L5 vertebral trabeculae (Tb) in OA and sham rats. Calcein as a calcium-chelating agent was injected into all rats at 6 and 1 days prior to tissue collection. Therefore, calcein signals on a bone-forming area appeared as double fluorescent lines (*arrows*), and the space between the two lines was the newly mineralized bone during the 5-day period. Ma designates marrow cavity. Bars, 100 µm. Bone formation-related parameters in OA and sham rats at 20 weeks after OA induction as determined by bone histomorphometry. (**B**) Osteoblast surface (Ob.S) normalized by bone surface (BS), (**C**) mineralizing surface (MS) normalized by BS, (**D**) double-labeled surface (dLS) normalized by BS, (**E**) mineral apposition rate (MAR), (**F**) bone formation rate (BFR) normalized by BS. Numbers of animals in each group are shown in parentheses. *P < 0.05 vs. sham group.

Interestingly, histomorphometric analyses of non-weight-bearing L5 vertebrae showed similar changes at 20 weeks after OA induction. A small decrease in bone volume (BV) without any changes in trabecular number (Tb.N), trabecular thickness (Tb.Th), or trabecular separation (Tb.Sp) was observed. Similar to the tibia, 20 weeks of OA increased the active erosion surface (aES) and osteoid volume (OV) but not osteoid thickness (O.Th) and osteoclast surface (Oc.S) in L5 vertebrae (Fig. 6). Although L5 vertebrae was not directly involved in OA development in the knee joint, dynamic bone turnover was also impaired by OA. Consistent with tibial analysis, 20 weeks OA impaired osteoblast activities as indicated by a decrease in bone formation rate (BFR), thereby resulting in decreases in mineralizing surface (MS) and double-labeled surface (dLS; Fig. 7).

## Discussion

ACLT in rodents is a standard OA induction procedure widely used to mimic post-traumatic OA in humans. The injuries of periarticular and articular soft tissues, including joint capsule, ligaments, and menisci cause joint instability, which in turn, leads to development of post-traumatic OA in humans^18^. The OA severity in the rodent models is dependent on the degree and duration of joint instability. It has been reported that ACLT alone led to mild OA, whereas ACLT together with medial meniscectomy caused moderate OA^19^. Therefore, ACLT was used in this study to induce mild knee OA that did not significantly limit animal’s locomotion. Otherwise, an impaired locomotion or a decrease in physical activity could indirectly affect bone remodeling.

Normally, calcium and bone metabolism are modulated by a number of factors, including hormones (e.g., estrogen, parathyroid hormone and prolactin), underlying diseases and pro-inflammatory cytokines^20–24^. It has been known that chronic inflammation due to OA could elevate both local and systemic proinflammatory cytokine levels^2,25^, some of which (e.g., IL-1, IL-6 and TNF-α) are capable of suppressing osteoblast function while accelerating osteoclast function^3,22–24^. There were reports in ACLT-induced OA rats that serum IL-1β and IL-6 levels were significantly elevated by two and fourfold, respectively^11,13^. For example, Ferrándiz et al. demonstrated that IL-1β level in 2-month-old ACLT rats was significant higher than that of sham-operated rats from ~ 110 to ~ 200 pg/mL^11^. Measurements of bone turnover markers revealed that knee OA was associated with increased c-telopeptide of type 1 collagen (CTX-1) and alkaline phosphatase levels^26^. We thus hypothesized that OA from ACLT would lead to bone loss in both local sites (i.e., tibiae) and distant sites (i.e., vertebrae).

Regarding the BMD analysis, the OA rats exhibited significant reduction in femoral trabecular vBMD in OA-operated leg of young adult (10 weeks), but not in mature adult (20 weeks) OA rats, whereas vBMD of tibia and L5 vertebrae did not change in either age groups (Fig. 3). This suggested that OA-associated low vBMD was more likely to be associated with weight-bearing bone, but not non-loading bone such as L5 vertebrae. The present finding was consistent with report by Ding et al. that decreased BMD predominantly occurred in hip bone^27^, but not spine in aged patients with radiographic knee OA. A reduction in trabecular vBMD in early OA was not uncommon. Patients with severe knee joint inflammation and progressive loss of articular cartilage were found to have a decreased femoral neck BMD as well as a decrease in ipsilateral proximal femur BMD that worsened with increased severity of knee OA^28^. It was possible that this reduction in BMD of proximal femur which was the main weight-bearing bone, was caused by reduced physical activity due to OA-associated joint pain^29^.

Bone histomorphometry analysis of tibial metaphysis in 20-week OA rats further revealed an increase in osteoclastic bone resorption together with increases in osteoid volume and thickness as compared with their intact legs (Fig. 4). Regarding the adaptive bone remodeling, one important factor that modulates the process is the magnitude of mechanical loading on bone. The mechanical loading is sensed by osteocytes, which in turn transfer signals to osteoblasts, resulting in either net bone gain or loss^30^. When mechanical loading is decreased or absent, osteocytes signal osteoclastogenesis indirectly via osteoblasts and subsequently stimulate bone resorption^31,32^. A reduction in mechanical load on the OA leg, which occurred as a result of shifted pattern of weight-bearing away from the OA leg to prevent further joint damage and to alleviate joint pain, was possibly responsible for stimulating osteoclastogenesis and consequently increasing the erosion surface^15,33,34^.

While OA enhanced bone resorption and impaired the mineralizing process, it did not alter some microstructural parameters, such as trabecular thickness, number or separation (Fig. 4). It is not uncommon that OA could increase bone volume. Evidently, OA was associated with increased collagen content and reduced calcium-to-collagen ratio^35^, which explained why increased osteoid thickness was observed with unaltered bone volume (Fig. 4). It is known that subchondral bone and articular cartilage are functionally related. Early bone marrow lesions in the subchondral bone in OA has been reported to alter bone microstructure by increasing bone volume through increasing trabecular thickness but with a reduction in the hardness of bone^36,37^.

There was also evidence that bone marrow lesions partially resulted from an overexpression of transforming growth factor (TGF)-β in the subchondral bone^38^. Since the increased expression levels of TGF-β transcripts and proteins were found in OA bone, TGF-β was believed to be an important player in OA^39,40^. TGF-β not only plays a role in the regulation of articular chondrocyte hypertrophy and maturation during OA development, but it also manipulates subchondral bone cell behavior, for example during formation of osteophyte, which is a fibrocartilage-capped bony outgrowth at the margins of diarthodial joints^41^. In the process of osteophytic formation, TGF-β enhances proliferation and differentiation of mesenchymal stem cell-like periosteal lining cells into hypertrophic chondrocytes. The mature chondrocytes ultimately undergo apoptosis and are replaced by osteoblasts and osteoclasts from bone marrow followed by angiogenesis, and then osteophyte becomes a part of the subchondral bone^41^. The role of TGF-β was strengthened by the finding that cells in the outer layers of osteophytes strongly expressed TGF-β1 and TGF-β3 in the murine model^42^.

Dynamic bone histomorphometric analysis suggested that OA also impaired osteoblast function as indicated by significant reductions in bone formation rate, mineral apposition rate, double-labeled surface and mineralizing surface (Fig. 5), accompanied with increases in osteoid volume and thickness (Fig. 4). The present data thus indicated an uncoupling of bone formation and resorption in the tibial metaphysis of OA-operated leg. It has been known that OA is associated with early loss of bone due to increased bone turnover^35^. In the late stage, there is a decrease in bone resorption with slight change in bone formation^35^. Thus, high bone resorption over bone formation as in the present study suggested that the 20-week OA was within the early stage rather than the late stage. Further experiments are required to determine the exact long-term effect of ACLT-induced OA on bone, and the longitudinal follow-up study must be extended, perhaps 20–30 weeks post-surgery.

Several factors that could be responsible for the decreases in osteoblast function were proinflammatory cytokines (e.g., IL-1β, IL-6, and TNF-α) as well as nitric oxide, the levels of which are normally elevated in the circulation or many tissues of OA individuals^25,43^. The high concentration of nitric oxide had inhibitory effect on osteoblast cell lineage and proliferation^44^. An increase in the production of inflammatory cytokines could also suppress osteoblast activity^44^. In addition, reports of hypomineralization of new collagen matrix in subchondral bone of femoral heads in OA patients and low serum calcium were consistent with the increased osteoid volume and osteoid thickness^45–47^.

Similar to the tibial metaphysis, static and dynamic bone histomorphometric analyses of non-weight-bearing L5 vertebrae of 20-week OA rats showed that OA did not affect trabecular number, thickness, or separation. In contrast to that observed in the tibiae, L5 vertebrae showed a small decrease in bone volume that corresponded with increased active erosion surface and osteoid volume. Osteoid thickness and osteoclast surface in OA-L5 vertebrae were increased albeit insignificantly (Fig. 6). Generally, dynamic bone histomorphometry of OA-L5 vertebrae showed similar pattern of skeletal changes that seen in tibial metaphysis (Fig. 7). Although mineral apposition rate in L5 vertebrae of OA rats was lower than that of sham-operated rats, the decrease was not statistically significant (Fig. 7). Smaller impact of OA on L5 vertebrae seen in the present study was also reported in other injury models, such as spinal cord injury that induced remarkable microstructural changes in weight-bearing tibiae, but not in non-weight-bearing lumbar vertebrae^48^. The functions of osteoblasts and osteoclasts in non-weight-bearing bone appeared to be less affected by a reduction in mechanical loading due to the OA-associated decrease in physical activity. Nevertheless, the presence of vertebral defects related to uncoupled osteoblast-osteoclast function has suggested that injury-induced knee OA was able to damage trabecular sites located distant from the injured knee. Perhaps, interventions that alleviate systemic proinflammatory cytokine burst might be beneficial for the OA patients.

In conclusions, knee OA induction by ACLT in young growing rats was shown to induce greater trabecular bone loss in the weight-bearing bone (tibiae) than in non-weight-bearing bone (L5 vertebrae) during OA progression. Bone loss was apparently caused by the enhanced bone resorption and decreased bone formation. Regarding the limitation, since this study was focused only in female rats owing to greater prevalence of OA and osteoporosis than males, whether similar trend occurs in male rats remains elusive. Nevertheless, our findings directly support concerns regarding the association between impact of repetitive physical activities such as in certain sports and the high risk of cartilage and joint injuries and the consequent bone loss.

## Supplementary information

Supplementary Information
