# Supplementary material for: Knee osteoarthritis in young growing rats is associated with widespread osteopenia and impaired bone mineralization
Source: Sci Rep. 2020 Sep 15;10:15079. doi: 10.1038/s41598-020-71941-8 (PMC7494904; doi:10.1038/s41598-020-71941-8)
Supplement: Supplementary file 1 — Supplementary Information [file 41598_2020_71941_MOESM1_ESM.pdf]

Supplementary Information

Knee osteoarthritis in young growing rats is associated with widespread osteopenia and impaired bone mineralization

Supitra Namhong[1,2], Kannikar Wongdee[2,3], Panan Suntornsaratoon[1,2], Jarinthorn Teerapornpuntakit[2,4], Ruedee Hemstapat[5], Narattaphol Charoenphandhu[1,2,6,7]*

[1] Department of Physiology, Faculty of Science, Mahidol University, Bangkok, Thailand
[2] Center of Calcium and Bone Research (COCAB), Faculty of Science, Mahidol University, Bangkok, Thailand
[3] Faculty of Allied Health Sciences, Burapha University, Chonburi, Thailand
[4] Department of Physiology, Faculty of Medical Science, Naresuan University, Phitsanulok, Thailand
[5] Department of Pharmacology, Faculty of Science, Mahidol University, Bangkok, Thailand
[6] Institute of Molecular Biosciences, Mahidol University, Nakhon Pathom, Thailand
[7] The Academy of Science, The Royal Society of Thailand, Bangkok, Thailand

Correspondence to:

Narattaphol Charoenphandhu, M.D., Ph.D.
Department of Physiology
Faculty of Science, Mahidol University
Rama VI Road, Bangkok 10400
Thailand
Tel & Fax: +66-2-354-7154
E-mail: naratt@narattsys.com

Subchondral area in tibia

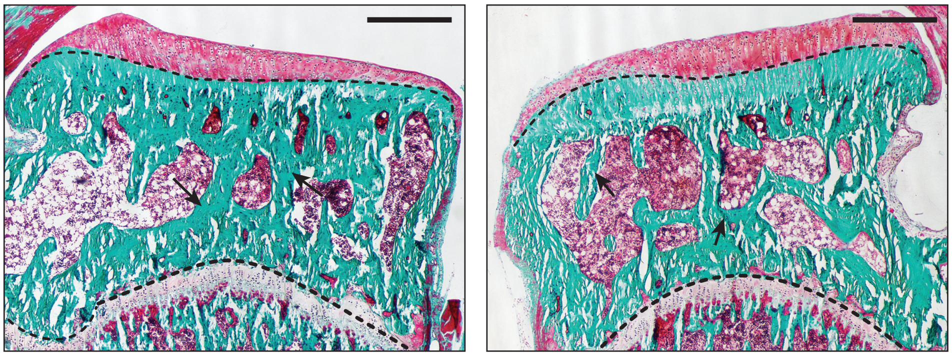

Intact OA

Supplementary Figure S1: Namhong et al.

Figure S1: Representative photomicrographs of Goldner's trichrome stained proximal tibia obtained from 20-week OA rat and its intact tibia. Areas within the dash lines were subchondral bone area. Mineralized trabeculae (arrows) and marrow cells were stained green and red, respectively. Bars, 1 mm.

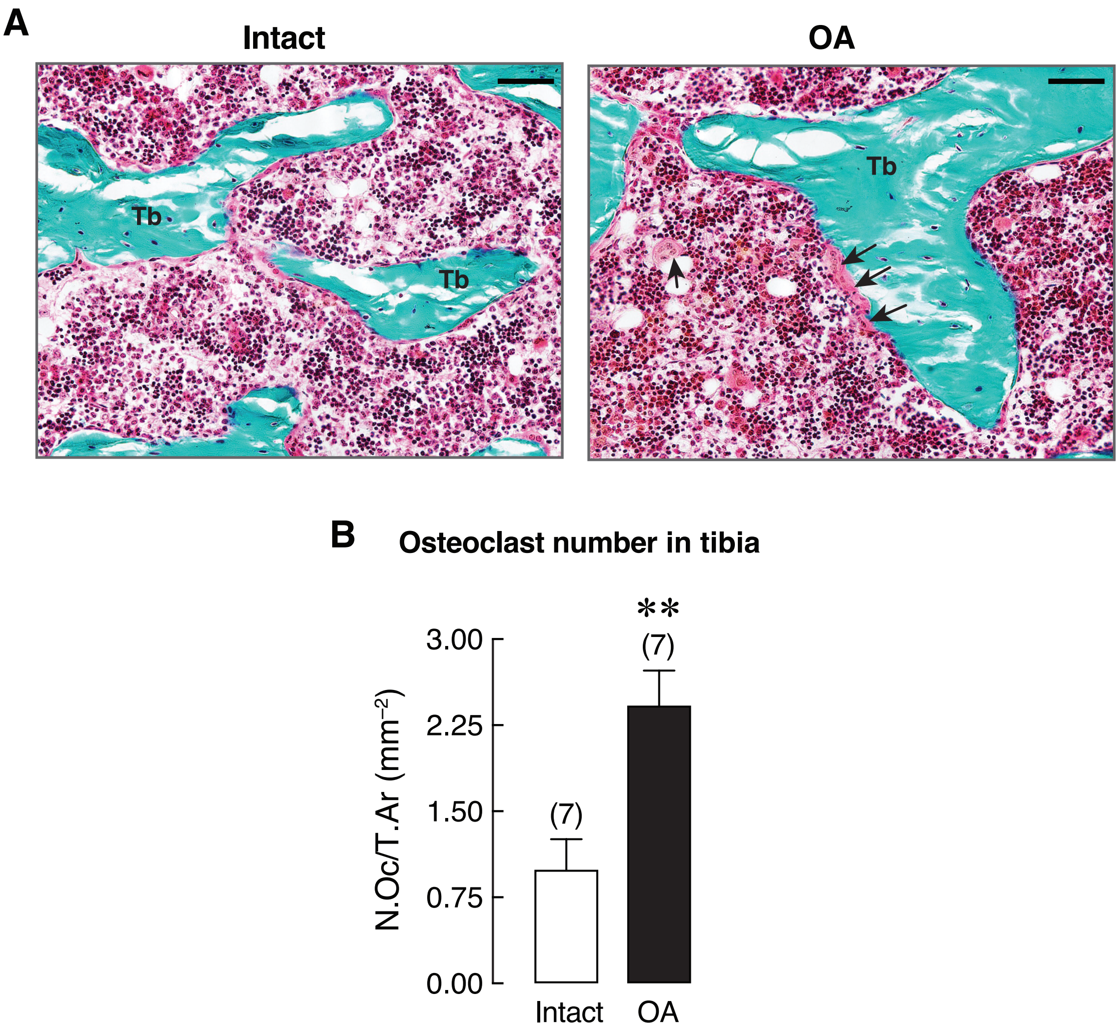

Supplementary Figure S2: Namhong et al.

Supplement Figure S2: (A) Representative photomicrographs of Goldner's trichrome stained tibia obtained from 20-week OA rat and its intact tibia. Mineralized trabeculae (Tb) and marrow cells active osteoclasts were localized on the bone surface. Bars, 50 µm. (B) Osteoclast number (N.Oc) normalized by tissue area (T.Ar). Numbers of animals in each group are shown in parentheses. $**P < 0.01$ vs. intact group.
